# Abnormalities of the oculomotor function in type 1 diabetes and diabetic neuropathy

**DOI:** 10.1007/s00592-022-01911-1

**Published:** 2022-06-22

**Authors:** Francesca D’Addio, Ida Pastore, Cristian Loretelli, Alessandro Valderrama-Vasquez, Vera Usuelli, Emma Assi, Chiara Mameli, Maddalena Macedoni, Anna Maestroni, Antonio Rossi, Maria Elena Lunati, Paola Silvia Morpurgo, Alessandra Gandolfi, Laura Montefusco, Andrea Mario Bolla, Moufida Ben Nasr, Stefania Di Maggio, Lisa Melzi, Giovanni Staurenghi, Antonio Secchi, Stefania Bianchi Marzoli, Gianvincenzo Zuccotti, Paolo Fiorina

**Affiliations:** 1 International Center for T1D, Pediatric Clinical Research Center Romeo ed Enrica Invernizzi, DIBIC, Università di Milano, Milan, Italy; 2 Division of Endocrinology, ASST Fatebenefratelli-Sacco, Milan, Italy; 3 Nephrology Division, Boston Children’s Hospital and Transplantation Research Center, Brigham and Women’s Hospital, Harvard Medical School, 300 Longwood Ave., Boston, MA 02115 USA; 4 Department of Pediatrics, Buzzi Children’s Hospital, Milan, Italy; 5 Neuro-Ophthalmology Center and Ocular Electrophysiology Laboratory, IRCCS Istituto Auxologico Italiano, Capitanio Hospital, Milan, Italy; 6 Clinica Oculistica, ASST Fatebenefratelli-Sacco, Università di Milano, Milan, Italy; 7 Transplant Medicine, IRCCS Ospedale San Raffaele, Milan, Italy; 8 Pediatric Clinical Research Center Romeo Ed Enrica Invernizzi, DIBIC, Università di Milano and Department of Pediatrics, Buzzi Children’s Hospital, Milan, Italy

**Keywords:** Type 1 diabetes, Oculomotor system, Eye movement tracking, Diabetic neuropathy

## Abstract

**Aims:**

Abnormalities in the oculomotor system may represent an early sign of diabetic neuropathy and are currently poorly studied. We designed an eye-tracking-based test to evaluate oculomotor function in patients with type 1 diabetes.

**Methods:**

We used the *SRLab—Tobii TX300 Eye tracker®*, an eye-tracking device, coupled with software that we developed to test abnormalities in the oculomotor system. The software consists of a series of eye-tracking tasks divided into 4 classes of parameters (Resistance, Wideness, Pursuit and Velocity) to evaluate both smooth and saccadic movement in different directions. We analyzed the oculomotor system in 34 healthy volunteers and in 34 patients with long-standing type 1 diabetes.

**Results:**

Among the 474 parameters analyzed with the eye-tracking-based system, 11% were significantly altered in patients with type 1 diabetes (*p* < 0.05), with a higher proportion of abnormalities observed in the Wideness (24%) and Resistance (10%) parameters. Patients with type 1 diabetes without diabetic neuropathy showed more frequently anomalous measurements in the Resistance class (*p* = 0.02). The classes of Velocity and Pursuit were less frequently altered in patients with type 1 diabetes as compared to healthy subjects, with anomalous measurements mainly observed in patients with diabetic neuropathy.

**Conclusions:**

Abnormalities in oculomotor system function can be detected in patients with type 1 diabetes using a novel eye-tracking-based test. A larger cohort study may further determine thresholds of normality and validate whether eye-tracking can be used to non-invasively characterize early signs of diabetic neuropathy.

*Trial:* NCT04608890.

**Supplementary Information:**

The online version contains supplementary material available at 10.1007/s00592-022-01911-1.

## Introduction

Diabetic neuropathy is responsible for a substantial number of hospitalizations and for reduction in the quality of life of patients with long-standing type 1 diabetes [1, 2]. The diagnosis of diabetic neuropathy encompasses a wide variety of clinical syndromes [3], which may affect distinct areas of the nervous system [3–5], and is primarily clinical, with poorly standardized criteria [6]. Indeed, the prevalence of diabetic neuropathy ranges from 10 to 90% in clinical studies [1, 2, 7], depending on the methods and criteria used [8], and more than one third of physicians miss the diagnosis even with overt diabetic neuropathy [9, 10]. Moreover, up to 50% of patients with diabetic neuropathy display no clear clinical symptoms [11], particularly in the case of adolescents and children with type 1 diabetes [12, 13]. Neuropathies may be either sensory or motor and may involve primarily large or small nerve fibers [4]. Small nerve fiber damage generally precedes large nerve fiber impairment, but abnormalities in small nerve fibers are difficult to study and to detect [9, 14]. Indeed, the oculomotor system, which primarily consists of small nerve fibers [15], is likely altered early in patients with type 1 diabetes and diabetic neuropathy; however, this hypothesis has never been studied due to the lack of non-invasive tests [4]. Therefore, little is known regarding the presence or effect of abnormalities in the oculomotor system in type 1 diabetes [16, 17]. Of particular note, oculomotor dysfunction has been observed in several neurodegenerative conditions, and eye-tracking technology has been recently studied and employed in Parkinson’s disease, Alzheimer’s disease, ALS and in some types of dementia to track disease onset and progression [18, 19]. Eye-tracking measures the point of gaze and/or the movement of the eye with respect to the head [20], and the eye tracker device analyzes both eye position and eye movement [20]. In this study, we explored whether the oculomotor system is functionally altered in patients with long-standing type 1 diabetes as compared to healthy subjects using a novel eye-tracking-based test. We hypothesize that patients with type 1 diabetes have a decreased capacity for eye movement, both in velocity and precision, and that these abnormalities are enhanced in patients with diabetic neuropathy.

## Methods

### Study population

Thirty-four patients with type 1 diabetes (T1D) and 34 healthy subjects were enrolled in this cross-sectional observational non-randomized study and recruitment lasted for 3 months (Table 1, Supplemental Fig. S1). T1D subjects were all receiving intensive insulin treatment at the time of enrollment in the study, while the group of healthy subjects was not being administered any medication and had normal glycometabolic control. 3/20 T1D subjects were on Levothyroxine treatment (Eutirox 25 mg *n* = 2, 125 mg *n* = 1) and 1/20 received statins. Alcohol consumption was not reported in any patient. Individuals with diabetic retinopathy and/or selected ocular disease (e.g., myopia > 6 diopters, best corrected visual acuity < 0.5 Snellen, corneal scar, strabismus or eye movement limitation) were not included. Previous history of optic neuropathy, uveitis, any vascular retinal disease, retinal detachment and previous microvascular cranial nerve palsy, were considered as exclusion criteria (Supplemental Fig. S1). All subjects provided informed consent before study enrollment. Studies not included in the routine clinical follow-up were covered by an appropriate Institutional Review Board approval.

**Table 1:** Demographic and clinical characteristics of patients enrolled in the study

	Healthy subjects (*n* = 34)	Type 1 diabetes patients (*n* = 34)	*P* value
Sex (M/F) – n	11/23	22/12	0.01
Age (y) – mean ± SEM	37.8 ± 1.7	32.2 ± 2.1	ns
BMI (Kg/m^2^) – mean ± SEM	22.3 ± 0.5	23.4 ± 0.5	ns
Duration of T1D (y) – mean ± SEM	N/A	18.2 ± 9.1	–
HbA1c at test (%) – mean ± SEM	N/A	7.8 ± 0.2	–
HbA1c at test (mmol/mol) – mean ± SEM	N/A	63.8 ± 2.4	–
HbA1c average (%) – mean ± SEM	N/A	8.0 ± 0.2	–
HbA1c average (mmol/mol) – mean ± SEM	N/A	62.5 ± 3.0	–
EIR (UI/Kg) – mean ± SEM	N/A	0.69 ± 0.03	–

n, Number; y, years; T1D, type 1 diabetes; BMI, body mass index; HbA1c, glycated hemoglobin; EIR, exogenous insulin requirement; SEM, standard error of the mean

### Diabetic neuropathy

Diabetic neuropathy was established based on clinical evaluation and according to clinical criteria described by the American Diabetes Association [12]. Among the 34 patients with T1D, 20 individuals did not have a clinical diagnosis of diabetic neuropathy, and 14 individuals had a clinical diagnosis of diabetic neuropathy. Diabetic peripheral neuropathy was assessed based on the Toronto Consensus [21] and classified as possible, probable and confirmed diabetic polyneuropathy (Supplemental Table S1).

### Neuro-ophthalmological evaluation

The study required all subjects to undergo a full neuro-ophthalmological evaluation (visual acuity, refractive error, slit lamp, intraocular pressure, posterior segment and ocular motility), as well as Spectral Domain Optical Coherence Tomography (SD-OCT, Optovue Freemont, CA) to assess macular and optic nerve structure. This evaluation was followed by the eye-tracking-based test. Briefly, for the ophthalmological evaluation, the best corrected visual acuity has been reported, while for the SD-OCT analysis, we considered the foveal thickness, the peripapillary retinal nerve fiber layer thickness and the macular ganglion cell complex thickness to assess macular and optic nerve damage, respectively.

### Eye-tracking system

**Fig. 1:**
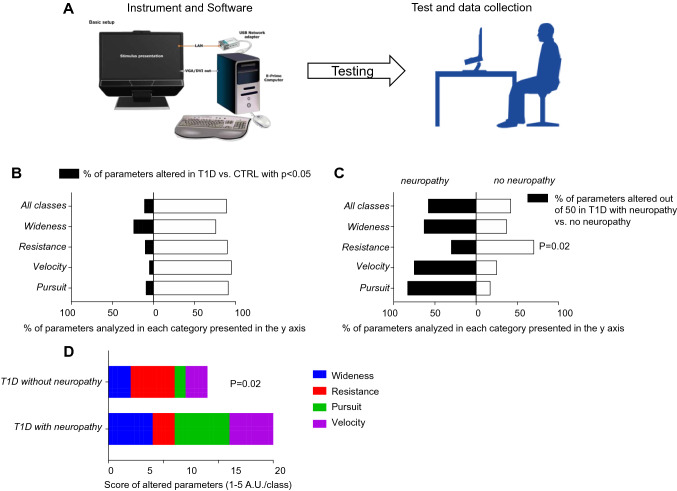
Cumulative distributions of all parameters analyzed using the eye-tracking-based test in healthy subjects and T1D patients with/without neuropathy. **a** Eye-tracking-based test and procedure. **b** Bar graphs depicting percentage of parameters analyzed in each class (Wideness, Resistance, Pursuit and Velocity) based on the statistically significant difference observed comparing CTRL versus T1D patients. White bars: % of parameters not altered (*p* > 0.05) when comparing T1D versus CTRL. Black bars: % of parameters altered when comparing T1D vs. CTRL (*p* < 0.05). **c** Bar graphs representing percentage of parameters altered (*n* = 50, *p* < 0.05) in patients with T1D and grouped by presence/absence of diabetic neuropathy. Black bars: % of parameters altered in T1D patients with neuropathy versus T1D patients without neuropathy (*p* < 0.05). **d** Summarizing score (1–5) evaluating proportion of altered parameters analyzed in each class (Wideness, Resistance, Pursuit and Velocity) in patients with T1D and grouped by presence/absence of diabetic neuropathy. CTRL, healthy subjects; T1D, patients with type 1 diabetes

Eye-tracking analysis was conducted in three groups of patients which included healthy subjects, T1D patients with neuropathy and without neuropathy. An eye-tracker is a device for measuring eye position and eye movement. We developed a test based on eye movement tracking for early, non-invasive detection of diabetic neuropathy. The SRLab—Tobii TX300 Eye tracker*®*, an eye-tracking device, is coupled with a software presenting a series of images/videos on the screen to test the velocity and accuracy of gaze movement. The eye-tracking tasks are divided into 4 classes: Resistance, Wideness, Pursuit and Velocity, which together aim to evaluate both smooth and saccadic movement in different directions. The SRLab—Tobii TX300 Eye tracker® uses an innovative technology that allows the study of eye movement without the need to be in close contact with the eyes [22]. The tracker is positioned below a screen on which stimuli are presented, and it can compensate for head movement and adapt to different eye types as well as glasses. The screen size is of 23.8″ size, and the resolution is of 1920 × 1080 pixels. The eye tracker is placed so that the gaze angle did not exceed ~ 35° to any point on the screen. The luminance (white) is of 300 cd/m^2^. Stimuli points were presented on a black background so as not to influence ambient light conditions. Testing was also done with ambient light at a level deemed ‘normal’ office lighting where the background is changed to white with black stimuli points. The distance from the person’s eyes to the eye tracker is approximately ~ 65 cm (26″). The sampling rate of the eye-tracking was 300 Hz and data were acquired binocularly. The tracker is connected to a computer with the Tobii StudioTM Eye Tracking Software®, which records patient data (Fig. 1a). We developed and combined a series of visualization stimuli, some of which already employed in other disease, to evaluate a subject’s ability to identify and follow a target moving at various positions and at different velocities (Supplemental Fig. S2). The stimuli employed to trigger specific eye movements followed by the eye-tracking device are divided into 4 classes:

1. Resistance: tests are divided into 17 eye-tracking tasks and in each task the visual stimulus consists of a target that must be found among different types and/or numbers of distractors. To pass to the next task, the subject must first identify the target. Differently from the other categories, each resistance task lasts as long as is needed for the subject to identify the target, instead of lasting for a predetermined amount of time (Supplemental Video 1). Therefore, we established two extra categories: TempiTask and TaskCompletati (see below).
2. Wideness: these tests are divided into 10 continuous movements with different directions; each movement is evaluated as the target moves around the screen for a predetermined amount of time (Supplemental Video 2).
3. Pursuit: 4 separate trajectories that originate from the edges of the screen and move inward, two horizontal and two vertical, each one repeated twice, evaluate how well the subjects can follow the target (Supplemental Video 3).
4. Velocity: the target, always originating from and returning to a central position, appears in 20 different areas of the screen and in all 4 quadrants of the visual field to evaluate saccadic movement (Supplemental Video 4).

Each class includes 8–10 categories in which different aspects of each parameter were analyzed as follows (Supplemental Tables S2–S5):

- FirsFixDur: The duration of the first fixation
- FixDur: Mean duration of fixations
- TimeFirsFix: Time from beginning of test to 1st fixation
- TotFixDur: Total duration of fixations
- TotVisDur: Total duration of visits
- VisitDur: Mean duration of visits
- FixCount: Total number of fixations
- VisitCount: Total number of visits
- TaskCompletati: Number of completed tasks (this is present only in Resistance)
- TempiTask: Time to complete a task (this is present only in Resistance)

Each stimulus was evaluated by the time necessary to identify the target, the time spent fixing target and the number of times in which the gaze passed over the target, for a total of 8 categories for each class, plus 2 extra categories for resistance (time to identify the target, number of targets identified). To fully analyze the test results, the following definitions were established:

- Fixation: the gaze remains on the target area (AoI, area of interest) for > 60 ms
- Visit: the gaze passes over the AoI, but remains for < 60 ms

All data were collected and analyzed by using the Tobii StudioTM Eye Tracking Software (Tobii Pro SDK) and transferred to Excel for further analysis. We also analyzed the proportion of altered eye movement in each class, as defined by statistically significantly different parameters in T1D patients with neuropathy and without neuropathy as compared to healthy subjects, using a categorical scoring system comprising 5 major points as follows: 1, less than 20% of parameters altered; 2, from 20 to 40% of parameters altered; 3, from 40 to 60% of parameters altered; 4, from 6 to 75% of parameters altered; 5, more than 75% parameters altered. Therefore, the higher the score the higher is the proportion of altered eye movement in the group of subjects included in the analysis.

### Statistical analysis

Continuous variables are presented as mean ± standard error (SEM) and compared with a two-tailed Student’s *t*-test for unpaired data (2 groups) or a one-way analysis of variance (3 or more groups), while the Mann–Whitney *U* test (2 groups) or the Kruskall-Wallis test (3 or more groups) was used if no normal distribution was evident (D’Agostino&Pearson/Shapiro–Wilk test). *P* value was adjusted for multiple testing by controlling the False Discovery Rate (Benjamini e Yekutieli, 2001) and/or by Sidak/Dunn’s test. Categorical variables were compared using the Chi-square/Fisher’s exact test as appropriate. A *p* value < 0.05 was considered statistically significant. Data analysis was performed using GraphPad Prism 7 (GraphPad Software, Inc., San Diego, CA).

## Results

### Patient characteristics

#### Healthy subjects and T1D patients

The demographic and clinical characteristics of the study population are summarized in Table 1. The mean age (± SEM) of healthy subjects was 37.4 ± 1.7 years as compared to 32.2 ± 2.1 years in patients with type 1 diabetes. In the healthy subject group, 11 out of 34 individuals included in the study were males as compared to 22 out of 34 in the T1D group. No differences were observed with regard to body mass index (BMI).

#### T1D patients with/without neuropathy

Mean age and gender did not differ between patients with T1D without neuropathy as compared to those with neuropathy. Duration of T1D, glycometabolic control at testing, BMI and exogenous insulin requirement were comparable as well (Table 2). Hypoglycemia was detected only in few T1D patients, with severity and frequency comparable in those with or without neuropathy (Table 2). Of the 14 patients diagnosed with diabetic neuropathy, 2 had abnormalities of nerve conduction detected at the electrophysiological test, 8 had neuropathic symptoms and decreased distal sensations and 4 had neuropathic sensory symptoms.

**Table 2:** Demographic and clinical characteristics of T1D patients enrolled in the study

	T1D without neuropathy (*n* = 20)	T1D with neuropathy (*n* = 14)	*P* value
Sex (M/F) – n	12/8	10/4	ns
Age (y) – mean ± SEM	33.8 ± 2.7	28.9 ± 3.2	ns
BMI (Kg/m^2^) – mean ± SEM	23.5 ± 3.4	24.1 ± 2.1	ns
Duration of T1D (y) – mean ± SEM	18.2 ± 9.1	15.7 ± 11.9	ns
HbA1c at test (%) – mean ± SEM	7.9 ± 1.4	7.3 ± 0.8	ns
HbA1c at test (mmol/mol) – mean ± SEM	64.2 ± 3.9	58.0 ± 2.6	ns
HbA1c 12 month-average (%) – mean ± SEM	8.3 ± 1.2	7.2 ± 0.3	0.02
HbA1c 12 month-average (mmol/mol) – mean ± SEM	66.3 ± 3.2	57.3 ± 1.9	0.02
EIR (UI/Kg) – mean ± SEM	0.70 ± 0.04	0.67 ± 0.08	ns
Hypoglycemia (Y/N) – n	3/17	1/13	ns

n, Number; y, years; BMI, body mass index; HbA1c, glycated hemoglobin; EIR, exogenous insulin requirement; SEM, standard error of the mean

#### Neuro-ophthalmological evaluation

No differences were observed between the groups in the analysis of best-corrected visual acuity and of the SD-OCT parameters when measuring foveal thickness, peripapillary retinal nerve fiber layer thickness and macular ganglion cell complex, thus indicating that none of the patients included in the study displayed structural signs of maculopathy or optic neuropathy (Table 3).

**Table 3:** Ophthalmological characteristics of patients enrolled in the study

	Healthy subjects (*n* = 30)	T1D without neuropathy (*n* = 20)	T1D with neuropathy (*n* = 14)	*P* value
BVCA OD – mean ± SEM	1.0 ± 0.1	1.0 ± 0.1	1.0 ± 0.6	*ns; °ns; §ns
BVCA OS – mean ± SEM	1.0 ± 0.1	1.0 ± 0.1	1.0 ± 0.1	*ns; °ns; §ns
Fovea OD (um) – mean ± SEM	243 ± 3	249 ± 4	244 ± 10	*ns; °ns; §ns
Fovea OS (um) – mean ± SEM	244 ± 4	246 ± 4	245 ± 10	*ns; °ns; §ns
RNFL OD (um) – mean ± SEM	107 ± 2	108 ± 2	109 ± 4	*ns; °ns; §ns
RNFL OS (um) – mean ± SEM	109 ± 2	106 ± 2	108 ± 3	*ns; °ns; §ns
GCC OD (um) – mean ± SEM	97 ± 1	96 ± 1	96 ± 2	*ns; °ns; §ns
GCC OS (um) – mean ± SEM	96 ± 1	97 ± 1	96 ± 2	*ns; °ns; §ns

T1D, Type 1 diabetes; BVCA, best-corrected visual acuity; RNFL, retinal nerve fiber layer thickness; GCC, ganglion cell complex thickness; SEM, standard error of the mean

*Healthy subjects versus T1D without neuropathy; Healthy subjects versus T1D with neuropathy; §T1D without neuropathy versus T1D with neuropathy

### Eye-tracking analysis

#### Overall study population

A total of 474 parameters were evaluated in the eye-tracking analysis, and 11% (50) of these parameters were significantly altered in patients with T1D with/without neuropathy (Fig. 1b), without a statistically significant effect of age and gender. Of the 4 major parameters evaluated by eye-tracking, Wideness showed the highest proportion of alterations, 24%, with a statistically significant difference when comparing healthy subjects to T1D patients (Fig. 1b). Resistance parameters also showed a high proportion of significantly altered measurements, 10% in T1D patients, as compared to healthy subjects (Fig. 1b), while 5 and 10% of parameters were significantly different in the analysis of Velocity and Pursuit, respectively (Fig. 1b).

### Sub-analysis

**Fig. 2:**
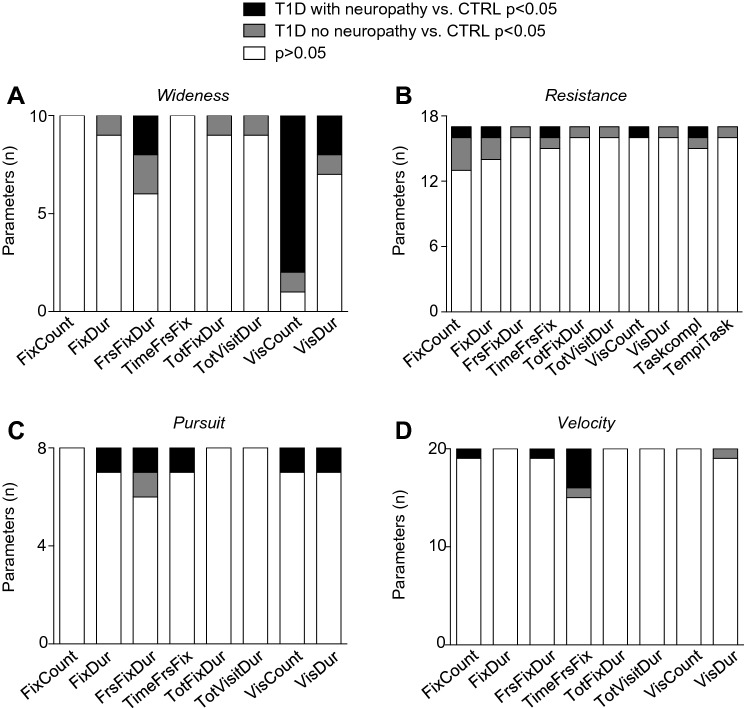
Cumulative distributions and proportions of all parameters analyzed using the eye-tracking-based test in healthy subjects and T1D patients grouped according to the presence/absence of diabetic neuropathy. **a–d** Bar graphs depicting number of parameters analyzed in the Wideness (**a**), Resistance (**b**), Pursuit (**c**) and Velocity (**d**) classes based on the statistically significant difference observed comparing CTRL versus T1D with/without neuropathy. CTRL, healthy subjects; T1D, patients with type 1 diabetes

Among the 50 parameters altered in the eye-tracking analysis, 28 (58%) were altered in patients with T1D and with neuropathy, while 22 (42%) were altered in the group of patients with T1D but without neuropathy (Fig. 1c). With regard to the major classes evaluated, Wideness was more altered although not achieving a statistically significant difference in T1D patients with neuropathy (12 out of 19, *p* = 0.2), while Resistance was mainly affected in T1D without neuropathy (Figs. 1c, d, 2a, b, 12 out of 17, *p* = 0.02). Analysis of Velocity showed fewer abnormalities in patients with T1D but without neuropathy (Figs. 1c,d, 2c), while Pursuit (Figs. 1c,d, 2d) showed differences mainly in patients with T1D and neuropathy. A score based on the analysis of differences between patients with T1D with or without neuropathy further confirmed the relevance of abnormalities detected in the Resistance class in the absence of diabetic neuropathy, and that of the Pursuit and Velocity classes in the presence of diabetic neuropathy (Fig. 1d).

#### Wideness

Interestingly, T1D patients, with and without neuropathy, required more time to localize and recognize the target as compared to healthy controls, with a comparable number of parameters altered (Fig. 2a and Table 4). With regard to the time to localize the target (i.e., FrsFixDur and VisDur), this appears increased in both T1D patients with and without neuropathy as compared to healthy subjects (Table 4). Conversely, an increased number of visits over the area of interest in 8 out of 10 movements tested and indicated as VisCount, was evident in T1D patients with neuropathy, but not in those without neuropathy, further suggesting that this alteration may be associated with established neuropathy in T1D (Table 4). This observation is also confirmed by a higher number of parameters related to Wideness analysis significantly altered in T1D patients with neuropathy as compared to those without neuropathy (Fig. 2a). Overall, several alterations in the widening of eye movements are evident in T1D patients as compared to healthy subjects, with a proportion of these alterations related to the presence of diabetic neuropathy.

**Table 4:** Major abnormalities detected by the eye-tracking-based test in patients with type 1 diabetes with/without neuropathy

	Healthy subjects	T1D without neuropathy	T1D with neuropathy	*P* value
*Wideness*				
FrsFixDur 9 (ms)	0.05 ± 0.01	0.04 ± 0.01	0.15 ± 0.03	*0.9; °0.007; §0.03
FrsFixDur 3 (ms)	0.01 ± 0.006	0.05 ± 0.01	0.06 ± 0.01	*0.01; °0.01; §0.4
VisDur3 (ms)	0.02 ± 0.007	0.05 ± 0.01	0.08 ± 0.01	*0.02; °0.01; §0.2
Viscount 9 (n)	0.8 ± 0.1	1.5 ± 0.4	1.9 ± 0.4	*0.09; °0.01; §0.1
Viscount 3 (n)	0.6 ± 1.8	2.1 ± 0.7	4.0 ± 1.3	*0.03; °0.002; §0.07
Viscount 4 (n)	1.1 ± 0.1	1.7 ± 0.2	4.7 ± 1.9	*0.08; °0.03; §0.2
Viscount 5 (n)	0.8 ± 0.1	1.4 ± 0.3	2.0 ± 0.4	*0.1; °0.02; §0.2
Viscount 7 (n)	1.1 ± 0.2	1.6 ± 0.3	4.0 ± 0.7	*0.09; °0.0006; §0.01
Viscount 10 (n)	1.5 ± 0.2	1.4 ± 0.3	14.8 ± 5.0	*0.9; °0.002; §0.004
*Resistance*				
FixCount oq60 (n)	0.6 ± 0.1	1.2 ± 0.1	1.2 ± 0.1	*0.02; °0.04; §0.6
FixDur oq40 DR (ms)	0.2 ± 0.02	0.4 ± 0.07	0.4 ± 0.08	*0.01; °0.01; §0.2
FrsFixDur Numeri Up R (ms)	0.2 ± 0.02	0.5 ± 0.1	0.4 ± 0.06	*0.03; °0.2; §0.4
TimeFrsFix oq80 (ms)	7.0 ± 1.2	15.7 ± 3.3	16.9 ± 5.1	*0.01; °0.01; §0.7
Fixdur Numeri Up R (ms)	0.2 ± 0.02	0.4 ± 0.06	0.3 ± 0.05	*0.005; °0.1; §0.1
Fixcount L80 03 (n)	0.7 ± 0.1	1.4 ± 0.1	1.0 ± 0	*0.005; °0.2; §0.2
*Pursuit*				
VisDur TopDown 1 (ms)	0.2 ± 0.02	0.2 ± 0.02	0.4 ± 0.007	*0.2; °0.0001; §0.0001
TimeFrsFix DxSx 1 (ms)	7.4 ± 0.5	6.8 ± 0.5	4.6 ± 0.3	*0.3; °0.009; §0.03
FrsFixDur TopDown 1 (msec)	0.1 ± 0.02	0.1 ± 0.02	0.2 ± 0.03	*0.2; °0.004; §0.01
*Velocity*				
FrsFixDur L near cross (ms)	0.2 ± 0.03	0.2 ± 0.03	0.4 ± 0.1	*0.1; °0.01; §0.006
Fixcount L far (n)	0.9 ± 0.1	1.2 ± 0.2	2.1 ± 0.4	*0.2; °0.008; §0.03

Data are expressed as mean ± SEM

T1D, Type 1 diabetes; L, left, R, right; DX, right; Sx, left; Fix, fixation; Frs, first; Dur, duration

*Healthy subjects versus T1D without neuropathy; Healthy subjects versus T1D with neuropathy; §T1D without neuropathy versus T1D with neuropathy

#### Resistance

Regardless of the presence of neuropathy, T1D patients required more time to localize and then recognize the target as compared to healthy subjects (Table 4). In particular, duration of fixation (FixDur) tended to increase in healthy subjects as compared to T1D patients without neuropathy, and then further increased in T1D patients with neuropathy in some types of movements (Table 4; Fig. 2b), while other parameters, particularly the number of fixations (FixCount), (Table 4), were mainly altered only in T1D patients without neuropathy, thus suggesting that T1D initially affects the number of fixation and the time needed to localize the target, and then in the presence of neuropathy there is a need to refocus every time the target. Overall, several alternations in the resistance are evident in T1D patients as compared to healthy subjects, with the majority being observed in the absence of diabetic neuropathy (52% vs. 12%, *p* = 0.02).

#### Pursuit

In the majority of cases, T1D patients with neuropathy required more time fixing the target as compared to healthy subjects or T1D patients without neuropathy, with FrsFixDur and VisDur being increased (Table 4). However, we observed that T1D patients with neuropathy required significantly less time than both the other groups in initially finding the target as shown by the reduction in TimeFrsFix Sx-Dx parameter (Table 4). This may be the cause for the increased fixation time: the target moves in a fixed time, if the patient finds it earlier, then he/she will consequently remain looking at the target for a longer period of time. Overall, the number of parameters significantly altered in patients with T1D and neuropathy was higher as compared to parameters altered in the T1D group without neuropathy, suggesting that Pursuit may be impaired in patients with T1D and established neuropathy (Fig. 2c). In summary, some abnormalities in the capability of the oculomotor system of tracking moving stimuli are evident in T1D patients as compared to healthy subjects, with the majority being observed in the presence of diabetic neuropathy.

#### Velocity

Interestingly, T1D patients with neuropathy had more difficulty in identifying a target after a quick saccadic movement instead of during smooth pursuit beginning on a fixed point (Table 4). Despite a reduction in the total amount of time spent on fixation for T1D patients with neuropathy as compared to that of healthy subjects, the latency of the first fixation was increased for some eye movements, thus suggesting this as a potential mechanism of compensation to allow for recognition of the target (Table 4). Moreover, the number of parameters significantly altered in T1D patients with neuropathy was higher as compared to parameters altered in T1D patients without neuropathy, further confirming the association between saccadic eye movement impairment and established neuropathy (Fig. 2d). In summary, rapid eye movements are impaired in T1D patients as compared to healthy subjects, with a higher number of alterations within the TimeFrsFix parameters observed in the presence of diabetic neuropathy.

## Discussion

The economic and healthcare burden associated with the development of diabetic neuropathy and its complications (i.e., autonomic dysfunction, neuropathic pain, foot ulcers) in patients with diabetes and particularly in those with T1D is well-known in the diabetic community [23–25]. Unfortunately, the diagnosis of this disease is extremely challenging [26], sometimes requiring the use of invasive methods (electromyography, nerve conduction studies, nerve/skin biopsies, quantitative sensory tests) [21, 27–29]. Recently, small nerve fiber damage and its non-invasive detection has gained interest and attention in the diagnosis of diabetic neuropathy, particularly in type 1 diabetes [30–32]. Our study demonstrated that the oculomotor system, primarily consisting of small nerve fibers, showed several abnormalities in patients with long-standing T1D when examined using a novel eye-tracking-based test. In particular, we observed that in patients with long-standing T1D, with or without diabetic neuropathy, a significant number of the eye-tracking parameters tested were altered as compared to healthy subjects, thus suggesting a subclinical involvement of eye movement system in diabetes. The clinical presentation of diabetic neuropathy involving the oculomotor system depends on altered function of the oculomotor, trochlear and abducens cranial nerves, which is often associated with double vision, restricted eye movement, mydriasis, and ptosis. In our study, patients with T1D displayed a higher proportion of abnormalities in the oculomotor system in the analysis of the Wideness and Resistance classes as compared to healthy subjects, while fewer differences were evident with regard to the Pursuit and Velocity classes. Among all the eye movement alterations observed in data collected from patients with T1D, half were found in patients with T1D with neuropathy and half in those without neuropathy, the latter potentially detecting early signs that precede the onset of the neuropathy. Interestingly, among all the classes analyzed by this novel eye-tracking-based test, T1D patients without neuropathy showed altered results in the Resistance class, while in T1D patients with neuropathy abnormalities in other classes were evident. These results may be due to the presence of a compensatory mechanism that alters normal tissue function prior to the onset of the disease but which then partially recedes [33], similar to the phenomenon of hyperfiltration in diabetic nephropathy [34, 35]. Indeed, in preclinical models, early signs of tissue alterations in the skin and Langerhans cells are evident in early stages of diabetes, but the diagnosis of diabetic neuropathy was established through the conventional technology at a later timepoint [36]. Hypoglycemia in T1D may also account for impaired eye movements [17], as those described in our eye-tracking-based test, but it was reported in very few patients, thus allowing to exclude it as the main mechanism involved. Alterations in glycemic control may affect the eye movement system as well, but the HbA1C levels measured at test in T1D patients with/without neuropathy were comparable, with different tasks being altered at the eye-tracking-based test. Conversely, the average HbA1C level observed in T1D patients with neuropathy at 12 months was lower, most likely due to the fact that the first line of intervention in patients with complications of diabetes, including diabetic neuropathy, consists in a strict control of the glycometabolic status in order to prevent progression of damage. We acknowledge that the small number of patients and the high number of parameters included in the analysis make interpretation of our sub analysis between patients with T1D and those with T1D without neuropathy exploratory, and further confirmatory studies are therefore required. Indeed, the number of patients with T1D and neuropathy, in this study is limited, and the study selection process that excluded retinopathy but included neuropathy may have also inadvertently defined a sub-population of patients with some predisposition for early development of neuropathy, who could be further investigated for other disease markers [37]. The clinical relevance of this non-invasive test to identify and screen altered eye movement patterns has been already established in other neurologic diseases (e.g., amyotrophic lateral sclerosis, Alzheimer’s disease, Parkinson’s disease, multiple sclerosis, and epilepsy) [38, 39], in which a cognitive disorder has been often recognized [19] and progression of the disease is hardly measurable [40]. Moreover, this method particularly recognizes alterations in rapid (saccades, velocity) and slow eye movements (smooth pursuit) and in some specific features (e.g., wideness and resistance), which are primarily under the control of the central nervous system. As patients with T1D may exhibit cognitive deficit as well in association with anomalous eye movements, our eye-tracking-based test, which is similar to that used in central nervous system disorders, may provide extra benefits. Our previous studies already demonstrated that alterations in central nervous system metabolism/function are evident in patients with T1D and may be associated with early cognitive decline and possibly senile dementia [41], thus our eye-tracking-based test identify an impairment of the oculomotor system function that may result from a central deficit and precede the establishment of overt neuropathy. Interestingly, the use of a video-based eye-tracking method as a low-cost alternative for detection of diabetic neuropathy in a small yet heterogenous population of diabetic subjects experiencing diabetic neuropathy and other diabetic complications (e.g., retinopathy, nephropathy), has been already tested with encouraging preliminary results [16, 42]. Keeping with this, our eye-tracking-based test was also effective in detecting anomalous eye movements in patients with T1D but without other diabetic complications (e.g., diabetic neuropathy, retinopathy and nephropathy), thus potentially acting as an early detection test for screening purposes. Our eye-tracking-based test consisted of a very rapid and non-invasive test, which lasted approximately 10 min and only required patients to sit relatively still and watch a computer screen. The eye-tracking device is small and can be connected to any computer, which is suitable for an ambulatory setting. Data are collected automatically by the Tobii StudioTM Eye Tracking Software and are easily transferred to Excel for analysis. The large amount of data collected using this test, which we acknowledge is a limitation due to the fact that it prevented complete analysis of each parameter in this preliminary study, may represent an enrichment strategy in future larger trials that may further benefit from the “big data” system supported by this technology. In summary, alterations in the oculomotor system can be detected in patients with long-standing T1D both with and without diabetic neuropathy. Larger studies are required to assess whether some parameters, such as VisCount within the wideness class and FixCount within the resistance class, may be selected in early non-invasive use of eye-tracking-based tests in patients with T1D to support the diagnosis of diabetic neuropathy and whether a threshold may be defined as compared to healthy subjects to facilitate diagnosis of the disease. Indeed, alterations observed in the VisCount within the wideness class in T1D patients, particularly in those with neuropathy, as compared to healthy subjects may suggest this as a confirmatory non-invasive measurement for established diabetic neuropathy, while abnormalities observed in the resistance class may be a sign of initial damage detectable in T1D patients early on and further disappearing when the neuropathy is established. With regard to pursuit and velocity classes, alterations observed mainly in T1D patients with neuropathy indicate that both rapid and slow eye movements are impaired and may represent a sign of established neuropathy in T1D, thus warranting to be included in the eye-tracking analysis to confirm the diagnosis of diabetic neuropathy. We acknowledge a number of limitations of this study, including the small number of patients enrolled in the subgroups, which may be due to the fact that we excluded T1D patients with retinopathy, which is often present in patients with T1D and particularly in those with neuropathy. Given the novelty of the technology explored here and the small number of patients included, normality ranges were not assessed in this analysis but may be further investigated in a larger study. Another potential limitation is that evaluation of the pursuit parameters involves central nervous system function, which could be impaired and may therefore alter results. Finally, the number of parameters included in the analysis is large and may not ensure rapid evaluation. Several testing parameters are redundant (i.e., FixDur, FrsFixDur or TotFixDur) and may be able to be combined to design a more rapid screening test. Nevertheless, after adjusting for multiple testing through the FDR, the majority of results were confirmed, with resistance class being altered mainly in T1D patients without neuropathy, while the classes of pursuit and velocity were mainly affected in patients with diabetic neuropathy. However, in view of the attention that big data analytics is gaining, the high number of parameters available in this eye-tracking test may also represent an asset for future studies aiming at evaluating the eye-tracking test as a potential non-invasive diagnostic tool in T1D and neuropathy.

## Supplementary Information

Below is the link to the electronic supplementary material.

Supplementary file1 (DOCX 402 kb)

Supplementary file2 (MOV 5361 kb)

Supplementary file3 (MOV 50394 kb)

Supplementary file4 (MOV 10602 kb)

Supplementary file5 (MOV 12831 kb)

## Data Availability

Some or all datasets generated during and/or analyzed during the current study are not publicly available but are available from the corresponding author on reasonable request.
