# Supplementary material for: Abnormalities of the oculomotor function in type 1 diabetes and diabetic neuropathy
Source: Acta Diabetol. 2022 Jun 22;59(9):1157–67. doi: 10.1007/s00592-022-01911-1 (PMC9329167; doi:10.1007/s00592-022-01911-1)
Supplement: Supplementary file 1 — Supplementary file1 (DOCX 402 kb) [file 592_2022_1911_MOESM1_ESM.docx]

**Abnormalities of the oculomotor function in type 1 diabetes**

Francesca D’Addio^1#^, Ida Pastore^2#^, Cristian Loretelli^1#^, Alessandro Valderrama-Vasquez^3^, Vera Usuelli^1^, Emma Assi^1^, Chiara Mameli^4^, Maddalena Macedoni^4^, Anna Maestroni^1^, Antonio Rossi^2^, Maria Elena Lunati^2^, Paola S. Morpurgo^2^, Alessandra Gandolfi^2^, Laura Montefusco^2^, Andrea Mario Bolla^2^, Moufida Ben Nasr^1,3^, Stefania Di Maggio^1^, Lisa Melzi^5^, Giovanni Staurenghi^6^, Antonio Secchi^7^, Stefania Bianchi^5^, Gianvincenzo Zuccotti^8^ and Paolo Fiorina^1,2,3*^

^1^International Center for T1D, Pediatric Clinical Research Center Romeo ed Enrica Invernizzi, DIBIC, Università di Milano, Milan, Italy; ^2^Division of Endocrinology, ASST Fatebenefratelli-Sacco, Milan, Italy; ^3^Nephrology Division, Boston Children’s Hospital and Transplantation Research Center, Brigham and Women’s Hospital, Harvard Medical School, Boston, MA, USA; ^4^Department of Pediatrics, Buzzi Children’s Hospital, Milan, Italy; ^5^Neuro-ophthalmology Center and Ocular Electrophysiology Laboratory, IRCCS Istituto Auxologico Italiano, Capitanio Hospital, Milano, Italy; ^6^Clinica Oculistica, ASST Fatebenefratelli-Sacco, Università di Milano, Milan, Italy; ^7^Transplant Medicine, IRCCS Ospedale San Raffaele, Milan, Italy; ^8^Pediatric Clinical Research Center Romeo ed Enrica Invernizzi, DIBIC, Università di Milano and Department of Pediatrics, Buzzi Children’s Hospital, Milan, Italy.

^*^Corresponding author; ^#^Co-first authors

**SUPPLEMENTAL FILES**

SUPPLEMENTAL FIGURE 1-2

SUPPLEMENTAL TABELS 1-5

SUPPLEMENTAL VIDEOS 1-4

**SUPPLEMENTAL FIGURES**

**Supplemental Figure 1**. **Flow-chart of the study**.

**Supplemental Figure 2**. **Eye-tracking based test screens**.

Anecdotal pictures of screens presented to patients during the analysis of the Resistance, Wideness, Pursuit and Velocity parameters.

**Supplemental Table 1. Clinical diagnosis of diabetic neuropathy in our study population.**

|  | **T1D with neuropathy n (%)** |
| --- | --- |
| Possible DSPN.  The presence of symptoms or signs of DSPN may include the following: symptoms–decreased sensation, positive neuropathic sensory symptoms. | 4 (29) |
| Probable DSPN.  The presence of a combination of symptoms and signs of neuropathy include any two or more of the following: neuropathic symptoms, decreased distal sensation, or unequivocally decreased or absent ankle reflexes. | 8 (57) |
| Confirmed DSPN.  The presence of an abnormality of NC and a symptom or symptoms or a sign or signs of neuropathy confirm DSPN. | 2 (14) |

**Abbreviations**: DSPN, distal symmetrical polyneuropathy; NC, nerve conduction; n, number.

**Supplemental Table 2**. **List of parameters analyzed by the eye-tracking-based test in the Wideness class.**

**Supplemental Table 3**. **List of parameters analyzed by the eye-tracking-based test in the Resistance class.**

**Supplemental Table 4**. **List of parameters analyzed by the eye-tracking-based test in the Pursuit class.**

**Supplemental Table 5**. **List of parameters analyzed by the eye-tracking based test in the Velocity class.**

**Supplemental Video 1. Wideness**

**Supplemental Video 2. Resistance**

**Supplemental Video 3. Pursuit**

**Supplemental Video 4. Velocity**
